# Multiple strand displacement amplification of mitochondrial DNA from clinical samples

**DOI:** 10.1186/1471-2350-9-7

**Published:** 2008-02-07

**Authors:** Samantha Maragh, John P Jakupciak, Paul D Wagner, William N Rom, David Sidransky, Sudhir Srivastava, Catherine D O'Connell

**Affiliations:** 1 Biochemical Science Division, National Institute of Standards and Technology, Gaithersburg, Maryland 20899, USA; 2 Cipher Systems, Crofton, Maryland, USA; 3 Cancer Biomarkers research Group, National Cancer Institute, Rockville, Maryland, USA; 4 Division of Pulmonary and Critical Care Medicine, NYU, School of Medicine, New York, USA; 5 Johns Hopkins University School of Medicine, 720 Rutland Ave, Baltimore, Maryland 21205, USA; 6 Tetracore, Inc., Rockville, Maryland, USA

## Abstract

**Background:**

Whole genome amplification (WGA) methods allow diagnostic laboratories to overcome the common problem of insufficient DNA in patient specimens. Further, body fluid samples useful for cancer early detection are often difficult to amplify with traditional PCR methods. In this first application of WGA on the entire human mitochondrial genome, we compared the accuracy of mitochondrial DNA (mtDNA) sequence analysis after WGA to that performed without genome amplification. We applied the method to a small group of cancer cases and controls and demonstrated that WGA is capable of increasing the yield of starting DNA material with identical genetic sequence.

**Methods:**

DNA was isolated from clinical samples and sent to NIST. Samples were amplified by PCR and those with no visible amplification were re-amplified using the Multiple Displacement Amplificaiton technique of whole genome amplification. All samples were analyzed by mitochip for mitochondrial DNA sequence to compare sequence concordance of the WGA samples with respect to native DNA. Real-Time PCR analysis was conducted to determine the level of WGA amplification for both nuclear and mtDNA.

**Results:**

In total, 19 samples were compared and the concordance rate between WGA and native mtDNA sequences was 99.995%. All of the cancer associated mutations in the native mtDNA were detected in the WGA amplified material and heteroplasmies in the native mtDNA were detected with high fidelity in the WGA material. In addition to the native mtDNA sequence present in the sample, 13 new heteroplasmies were detected in the WGA material.

**Conclusion:**

Genetic screening of mtDNA amplified by WGA is applicable for the detection of cancer associated mutations. Our results show the feasibility of this method for: 1) increasing the amount of DNA available for analysis, 2) recovering the identical mtDNA sequence, 3) accurately detecting mtDNA point mutations associated with cancer.

## Background

It is often the case that an insufficient quantity of DNA can be isolated from clinical specimens and controls for full genome analysis. The quantity of DNA is particularly limited in patient tumor specimens, most notably in early tumors with limited mass and therefore, insufficient DNA may be available to perform the multiple analyses required for full genome screening [1-4]. Whole genome amplification (WGA) methods have been developed to solve the problem of insufficient quantities of DNA [5,6]. Using these technologies, investigators have been successful in applying genome scanning technologies to patient cohort samples collected years ago that are not recoverable by other means [7]. WGA is useful for amplification of DNA from stored histology slides, tissue samples, and blood stains, including amplification from single sperm [8-10].

Two common methods used for WGA are multiple-displacement amplification (MDA) and library synthesis from fragmented genomic DNA (gDNA). MDA uses the highly processive Ø29 DNA polymerase and random exonuclease-resistant primers in an isothermal amplification reaction [11]; this method is based on strand-displacement synthesis [12,13]. A second commonly used method converts randomly fragmented gDNA into a library of DNA fragments of defined size; this library can be effectively amplified several thousand-fold using a high-fidelity DNA polymerase and can be re-amplified to achieve a final amplification of over a million fold without degradation of representation [14]. Both of these methods produce material suitable for downstream multiplex analyses [15,16].

An advantage of the MDA method is the generation of significant quantities of large fragments of amplified gDNA in a single step. The library-based method has the advantage that it enables the creation of whole-genome DNA libraries from degraded as well as intact DNA samples. However, care should be exercised to rule out locus and allele dropouts in the WGA product. For efficient amplification, the template should be a minimum of 2000 base pairs in length and optimally greater than 10,000. A minimum of 10 ng of DNA is required [17]. For the study described in this paper, the MDA method was used due to ease of use and perceived integrity of the circular mitochondrial DNA. The conclusions from these studies are that whole genome amplification provides a useful tool when there are limited quantities of DNA.

Although clinical testing of genomic DNA amplified with WGA techniques demonstrated excellent concordance for the detection of point mutations [18,19], WGA has not been widely accepted because of concerns about replication accuracy. Further, papers reporting the use of WGA techniques have focused on the evaluation of the technique rather than on the accuracy with patient specimens. There are also a variety of factors to note when comparing WGA studies, e.g. tissue acquisition, fixation, sectioning, etc [11]. Although mtDNA is more abundant than nuclear DNA, the same limitations are relevant with mtDNA.

Early detection of disease associated mutations frequently requires the analysis of matched body fluids samples. Often body fluids that are used to assess the presence of cancer in remote sights contain degraded DNA or very low quantity. To validate the usefulness of body fluids to potentially replace tumor tissue as a source of mtDNA, we identified a WGA system that fulfills our requirements with respect to product yield and quality and have optimized this WGA method for the entire human mitochondrial genome. Prior studies using WGA for mtDNA reported on the control region, which only accounts for 6% of the mitochondrial genome [20,21]. In our study, sequence concordance for native genomic DNA and WGA-amplified DNA was analyzed using the Affymetrix GeneChip^® ^mitochondrial CustomSeq^® ^resequencing microarray, versions 1 and 2. These arrays have increased sensitivity over traditional fluorescent DNA sequencing for the detection of mixed bases, or heteroplasmies [22]. Overall, the sequence identity between native and WGA material was 99.9995%. Further, for disease associated mutations (genetic differences between tumor tissue and matched blood samples) WGA correctly identified 100% of the mutations. Heteroplasmies were accurately detected using WGA. Our results show that WGA is a reliable method for amplifying mtDNA from body fluids (sputum), blood and tumor tissue.

## Methods

### DNA isolation: blood and matched tumor

Paired normal and tumor specimens were collected after surgical resection with prior consent from patients in the Johns Hopkins University Hospital. DNA samples were provided by Dr. David Sidransky at the Johns Hopkins School of Medicine. Samples were collected primarily from early stage (stage IA to IIB) tumors. DNA was extracted from micro-dissected tumor tissue obtained from cryostat-embedded snap-frozen sections. Tumor sections were digested with 1% SDS/Proteinase K, and DNA extracted by phenol, chloroform and ethanol precipitated. Tumor samples were obtained from males and females.

### DNA isolation: non cancer samples

DNA from blood and sputum from 8 individuals in Trizol^® ^Reagent was provided by Dr. William Rom New York University School of Medicine. The DNA was recovered using the manufacturer's protocol (Invitrogen Corp., Carlsbad, CA). Patients were considered cancer free based upon spiral CT analysis. MtDNA from three matched blood and sputum samples, and one un-matched blood and 4 unmatched sputums were analyzed from these eight individuals. The remaining samples from these individuals (4 blood samples and 1 sputum) could not be analyzed without WGA amplification.

### PCR amplification: Repli-g Whole Genome Amplification (WGA)

A REPLI-g midi kit (Qiagen Inc., Valencia, CA) was used to amplify total DNA. The REPLI-g kit amplifies isothermally at 30°C using Ø29 DNA polymerase with 3' → 5' exonuclease proofreading activity and multiple displacement amplification. The published "Whole Genome Amplification Using Purified Genomic DNA" protocol was followed. Briefly, buffers D1-denaturation buffer and N1-neutralization buffer were made in accordance with the protocol. Template DNA (2.5 μL), 2.5 μL of buffer D1 was added and incubated at room temp. for 3 minutes; then 5 μL of buffer N1 was added. Forty microliters of the replication mix (10 μL H_2_O, 29 μL REPLI-g Midi Reaction Buffer, 0.5 μL REPLI-g Midi DNA Polymerase) was added on ice to the denatured DNA for a total volume of 50 μL. The mix was incubated for 16 hrs at 30°C. Polymerase activity was stopped by a 5 minute incubation at 65°C. WGA DNA was then reamplified (1 μL WGA per PCR reaction) using the 3 MitoChip primer sets and the Affymetrix/JHMI protocol [23].

### Real-time PCR analysis

Quantities of the native template and WGA nuclear and mtDNA were measured by Real-Time PCR on a Chromo4 system (Bio-Rad Laboratories Inc., Hercules, CA). The TNF gene was used as a nuclear DNA marker and 12SrRNA was used as a mtDNA marker as described [24]. Briefly each PCR reaction contained: 12.5 μL 2x iQ SYBR Green Supermix (Bio-Rad), forward and reverse primers (125 nM final concentration), template DNA and water to a final volume of 25 μL. Thermal cycling conditions: (1 cycle) 95°C for 3 min, (45 cycles): 95°C for 30 sec, 61.5°C for 30 sec, 72°C for 30 sec, (1 cycle) 72°C for 10 min, melt curve 50°C to 105°C increasing at 1°C intervals with a 3 sec hold. The NIST Human DNA Quantitation Standard (SRM 2372) was used to calibrate the standard curve of the quantitative PCR assay. Quantitative PCR values were used to calculate the mass of nuclear DNA and mtDNA added to the WGA reaction, the amount of amplified nuclear and mtDNA after WGA, and the fold amplification of nuclear and mtDNA obtained by using WGA.

### PCR amplification

Three matched primer sets were used to amplify the DNA for hybridization onto the Affymetrix GeneChip^® ^mitochondrial CustomSeq^® ^Resequencing microarray. In our initial studies, version 1 arrays were used to analyze DNAs from 8 non-cancer individuals and 6 cancer patients. After the release of the version 2 array, DNAs from the 6 cancer patients were reanalyzed with the GeneChip^® ^Human Mitochondrial Resequencing Array 2. The primer sets together amplified the entire mitochondrial genome. Quality control, as outlined previously was followed [25]. Briefly, for the MitoChip, the mtDNA template (20 ng up to 10 μL), 0.75 μL of forward and reverse primers (10 μM), 0.5 μL LA Taq polymerase (TaKara), 5 μLPCR buffer, 8 μL dNTP (2.5 mM each) and dH_2_O up to a total reaction volume of 50 μL. Thermal cycling conditions were as follows: pre-amplification denaturation: (1 cycle), 94°C for 2 min; amplification (30 cycles): 94°C for 15 s; annealing and elongation, 68°C for 7 min; final elongation (1 cycle), 68°C for 12 min; 4°C hold. Amplification of PCR products was confirmed by gel electrophoresis on a 0.8% agarose gel. PCR amplification products were analyzed for quality and quantity by spectrophotometric methods as described in GeneChip CustomSeq™ Resequencing Array Protocol Version 2.

### PCR cleanup: MitoChip

PCR clean up was conducted using the QIAquick 96 well vacuum plate manifold and protocol [26]. DNAs were eluted in 65 μL of DNAse/RNAse free water.

### MitoChip protocol

The GeneChip^® ^CustomSeq^® ^Resequencing Array Protocol Version 2.0 was followed with a few modifications. Briefly, three amplicons representing the mitochondrial genome were pooled at equi-molar concentrations. The pooled PCR amplification products were fragmented, labeled, hybridized, washed and scanned. The total quantity of DNA applied to the array was 0.62 μg. Fragmentation of the pooled DNAs was conducted using 0.15 units of Fragmentation reagent (0.033 μL) per sample at 37°C for 35 minutes followed by 95°C for 15 minutes to inactivate the enzyme. The fragments were labeled with 30 units of TdT using the Affymetrix GeneChip^® ^DNA Labeling Reagent containing a proprietary biotinylated label. Labeling was carried out at 37°C for 90 minutes followed by 95°C for 15 minutes. The hybridization cocktail including the separately prepared control fragments were hybridized for 16 to 18 hours at 45°C rotating at 60 rpm. Arrays were scanned on a GeneChip^® ^Scanner 3000G7 Scanner, and analyzed with GeneChip^® ^Operating Software (GCOS v1.4) and GeneChip^® ^Sequence Analysis Software (GSEQ v4.0).

### MitoChip sequence interpretation

Final analysis of all data was conducted using Affymetrix software GCOS v1.4 and GSEQ v4.0 in addition to an in house analysis program. Probe intensities for each mutation reported by the software was examined on both forward and reverse stands. Heteroplasmies were only confirmed and reported if present on both strands.

## Results

**Figure 1:**
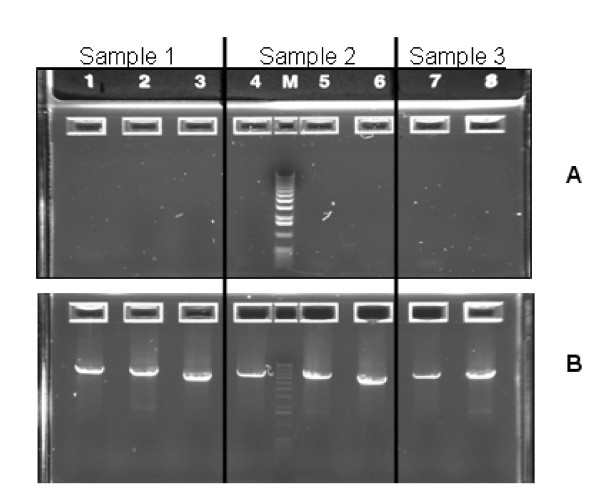
Gel image after PCR amplification of the three mtDNA primer sets used in this study. DNA from three samples (1–3) were amplified without (panel A) and after WGA (panel B), then separated on a 0.8% agarose E-Gel (Invitrogen). Lanes 1–3: sample 1, lanes 4–6: sample 2, and lanes 7–8: sample 3. For all three samples the PCR products are shown in the following order on the gel: 6185 bp product, 6015 bp product and 5063 bp product.

Sequencing of mtDNA from tumor, blood and body fluids from the same individuals was carried out to determine whether body fluids could be used to detect cancer. We were frequently unable to compare all three specimens as there was insufficient DNA available for sequencing in one or more samples [4,22,27]. However, WGA provided ample DNA for these experiments. Thirteen of twenty-four non-cancer specimens could not be amplified by PCR, but all twenty-four specimens were amplifiable after using WGA. This is shown in Figure 1 for three of thirteen non-cancer samples whose native DNA was not amplifiable by PCR (panel A), and PCR products of the correct length for the same specimens post WGA (panel B). To determine whether WGA altered the sequence of mtDNA, we compared mtDNA sequences in unamplified mtDNA (native) and WGA mtDNA for both cancer and non-cancer samples.

### Fold amplification of nuclear and mtDNA by WGA

Real-Time PCR measurements were used to determine the extent of WGA amplification of specimen DNAs. Because WGA amplifies total DNA present separate markers were used to determine quantities and fold amplification of nuclear (TNF) and mtDNA (12SrRNA). For all samples, there was at least a 120 fold amplification of nuclear DNA and 133 fold amplification of mtDNA after WGA; with WGA outputs of at least 880 ng of nuclear DNA and 1,260 ng of mtDNA (Table 1). The extent of amplification roughly correlates with the amount of input DNA in an inverse relationship, where the samples with less input DNA had higher fold amplification than the samples with higher amounts of input DNA.

**Table 1:** Real-Time PCR: nuclear and mtDNA native and WGA samples

	Native input nuclear DNA (ng)	Nuclear DNA in 50 uL WGA (ng)	Fold nuclear DNA amplification by WGA	mtDNA input nuclear DNA (ng)	mtDNA in 50 uL WGA (ng)	Fold mtDNA amplification by WGA	
CB 4	6.80E-01	2.80E+04	4.12E+04	1.28E+00	1.38E+04	1.08E+04	CB 4
CB 1	1.65E+00	1.98E+04	1.20E+04	2.38E+00	3.66E+04	1.54E+04	CB 1
CS 3	2.03E+00	1.07E+04	5.25E+03	3.50E+00	2.82E+03	8.05E+02	CS 6
CS 6	2.05E+00	8.38E+03	4.08E+03	7.65E+00	2.94E+03	3.84E+02	CS 8
CS 2	4.28E+00	1.11E+04	2.58E+03	8.12E+00	1.50E+04	1.85E+03	CS 1
CS 1	5.14E+00	3.47E+03	6.75E+02	8.13E+00	1.93E+03	2.38E+02	CS 3
CS 8	7.29E+00	6.15E+03	8.44E+02	1.29E+01	5.47E+03	4.24E+02	CS 2
CB 2	1.23E+01	6.37E+03	5.19E+02	1.26E+02	1.17E+05	9.24E+02	CS 5
CB 3	6.52E+01	7.82E+03	1.20E+02	2.15E+02	3.07E+04	1.43E+02	CB 2
CS 5	9.37E+01	2.83E+04	3.02E+02	2.92E+02	3.87E+04	1.33E+02	CB 3
CS 7	n/a	5.82E+03	n/a	n/a	3.06E+03	n/a	CS 7
							
PB 2	1.46E-01	9.06E+03	6.19E+04	0.04	8.33E+03	2.10E+05	PB 4
PB 4	1.56E-01	7.43E+03	4.75E+04	0.05	2.84E+03	6.08E+04	PB 2
PT 3	1.70E-01	8.82E+02	5.19E+03	0.26	1.26E+03	4.91E+03	PB 1
PT 2	2.26E-01	5.47E+03	2.42E+04	0.27	4.32E+03	1.60E+04	PB 3
PB 3	2.36E-01	2.95E+03	1.25E+04	0.41	8.03E+03	1.94E+04	PT 2
PB 1	4.77E-01	2.85E+03	5.97E+03	0.77	3.51E+03	4.58E+03	PT 3
PT 1	5.33E-01	4.41E+03	8.27E+03	1.45	4.08E+03	2.81E+03	PT 1
PB 5	n/a	4.59E+03	n/a	n/a	1.60E+04	n/a	PB 5

n/a: insufficient DNA present for assa

### Native vs. WGA mtDNA sequence: non cancer blood and sputum

**Figure 2:**
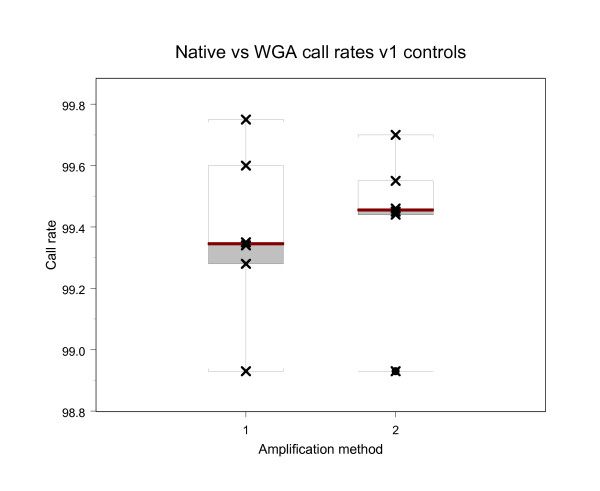
Boxplots of call rates obtained from version 1 mitochondrial resequencing arrays for 11 samples. (1) Native blood and sputum DNAs from non-cancer individuals and (2) WGA blood and sputum DNAs using the native DNAs in 1.

Native mtDNA and WGA mtDNA from three matched blood and sputum samples from non cancer individuals were sequenced on the version 1 Mitochip (n = 6). These DNA samples were isolated using a different method (Trizol) than that used for the cancer patient DNAs. There was no difference in the call rate of the chips for native mtDNA (99.38% +/-0.28) and WGA mtDNA (99.42% +/-0.26) (Table 2 and Figure 2). Boxplots are used to represent the call rate data [28]. The box represents the interquartile range of the data with the "wiskers" stretching from the box defining the maximum and minimum data points. The horizontal line within the box is the median point of the dataset. Of the six genomes compared, one homoplasmic difference was identified between the native and WGA sequences (Table 2) where the native sequence was a T and the WGA material was a C. Thus, sequence identity of 99.9994% for the 6 native and WGA samples was obtained.

**Table 2:** Controls: native vs. WGA

Controls	Native vs. WGA		
**Sample**	**Conflicts**	**Type**	**Position**

CB 1	0	n/a	
CB 2	1	Homo	T11471C
CB 3	0	n/a	
CB 4	0	n/a	
CS 1	0	n/a	
CS 2	0	n/a	
CS 3	0	n/a	
CS 5	0	n/a	
CS 6	1	Hetero	A10750R
CS 7	0	n/a	
CS 8	0	n/a	

* Control Blood + Control Sputum

The mtDNA sequences from an additional 5 samples (1 blood and 4 sputums) from non cancer individuals were also analyzed on the version 2 array. The sequence comparison of native and WGA mtDNA resulted in 99.9994% concordance between the sequences, with a discordance of callable bases of 0.00054%. For 1 nucleotide position in a single WGA sample, a heteroplasmy was detected that consisted of the native nucleotide A, plus a base transition G (Table 2).

### Native vs. WGA mtDNA sequence: blood and tumor patient samples

**Figure 3:**
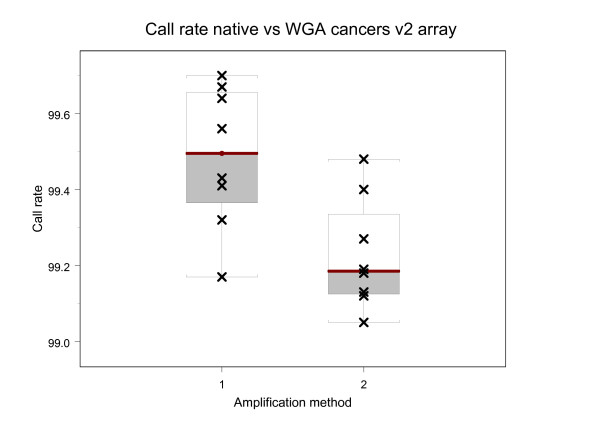
Boxplots of call rates obtained from version 2 mitochondrial resequencing arrays for 8 samples. (1) Native blood and tumor DNAs from Lung cancer patients and (2) WGA blood and tumor DNAs using the native DNAs in 1.

Blood and tumor samples from four lung cancer patients were amplified (without WGA) with three primer sets and sequenced on the version 1 mitochondrial resequencing array with an average sequence call rate of 99.61% +/-0.16. These same 8 samples were amplified (with and without WGA) and sequenced on the version 2 mitochondrial resequencing array. Mitochondrial sequence average call rates for native and WGA samples were 99.49% +/-0.19 and WGA 99.23% +/-0.15 respectively (Table 3, Figure 3). The comparison of native and WGA mtDNA sequences from five blood and three tumor samples (n = 8) identified a 99.995% concordance between native and WGA sequences and a discordance of callable bases of .00495%. There was no difference in the native and WGA mtDNA sequences for three of the samples. Five of the 8 samples had on average 2 heteroplasmy calls accounting for 0.005% sequence disagreement (Table 4). For twelve of the base call differences, there was a homoplasmic call in the native sample (the wildtype sequence information) and there was a heteroplasmy for the WGA sample. In one case there was the opposite of this, with a heteroplasmy detected in the native sample and a homoplasmic call agreeing with the reference sequence, tiled on the MitoChip, in the WGA sample.

**Table 3:** Patient samples: native vs. WGA

	Native vs. WGA	
**Sample**	**Conflicts**	**Type**

PB 1	1	Hetero
PB 2	2	Hetero
PB 3	3	Hetero
PB 4	3	Hetero
PB 5	0	n/a
PT 1	0	n/a
PT 2	4	Hetero
PT 3	0	n/a

* Patient Blood + Patient Tumor

**Table 4:** Discrepant calls

	Mito Pos	Ref	Native	WGA
PB1	8723	G	*	R
				
PB2	7553	A	*	R
	10450	T	*	Y
				
PB3	1350	G	*	R
	6365	T	*	Y
	12047	T	*	Y
				
PB4	189	A	*	R
	6365	T	*	Y
	14185	A	R	*
				
PT2	2783	A	*	R
	2966	T	*	Y
	6102	T	*	Y
	8725	A	*	R

### Comparison of version 1 and version 2 MitoChip arrays

Previous studies demonstrated that the Affymetrix version 1 CustomSeq^® ^Resequencing array for human mitochondrial DNA was an accurate and highly sensitive tool for mtDNA sequencing, particularly in detecting heteroplasmies [23]. However, this array contained only the mitochondrial DNA coding region (nts 577-16023 of the 16,568 bp genome). Subsequently, Affymetrix developed a second generation chip that contains the entire mtDNA genome. This second generation array was determined to have call rates comparable to the version 1 array, and a chip to chip error rate of 0.00328% with potentially higher sensitivity than the version 1 array due to the additional sequence coverage [29].

**Figure 4:**
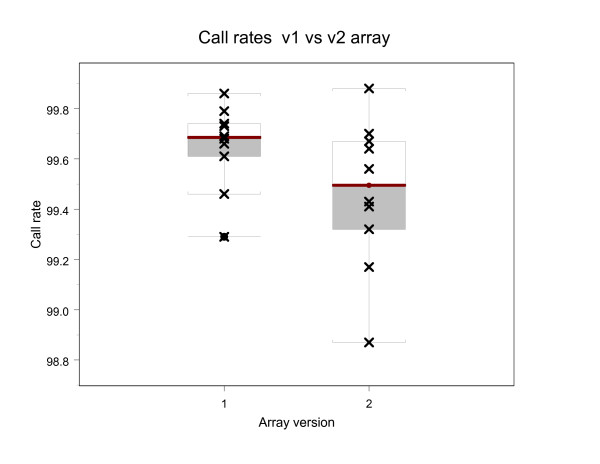
Boxplots of call rates for 10 native blood and tumor DNAs sequenced on both the version 1 and version 2 mitochondrial resequencing array. (1) Samples sequenced on version 1 array (2) Samples sequenced on version 2 array.

To verify that the sequence data obtained from version 1 and version 2 mitochondrial resequencing arrays are comparable, DNA s from 6 cancer patients were analyzed on both versions of the array. In total, 10 samples (6 bloods and 4 tumors) were sequenced on both arrays, as insufficient quantities of DNA were available to amplify 2 tumor samples. All sequences were first compared to the RCRS sequence to generate SNP reports. The reports for the same samples were compared for each version, and all different calls were evaluated. There was no significant difference between the average number of calls generated for each genome on the two arrays; 99.66% +/-0.15 for v1 arrays, and 99.47% +/-0.29 for v2 arrays (Table 5, Figure 4). The comparison showed that the sequences obtained from version 1 and version 2 arrays are 99.999% identical, with a total of 2 differences identified between the 2 versions of the array. The discordance between version 1 and version 2 chips of the same sample DNA was 0.00065%. As stated in the materials and methods, heteroplasmic calls were only made if the heteroplasmy was detected on both sense and antisense strands.

**Table 5:** Version1 vs Version 2 Arrays

	v1 vs. v2		
**Sample**	**Conflicts**	**Type**	**Position**

PB 1	0	n/a	
PB 2	0	n/a	
PB 3	0	n/a	
PB 4	1	hetero	A14185R
PB 5	0	n/a	
PB 6	1	hetero	C7256Y
PT 1	0	n/a	
PT 2	0	n/a	
PT 3	0	n/a	
PT 4	0	n/a	

## Discussion

The ability to detect cancer associated mutations in clinical samples is frequently limited by the quantity of DNA obtained from each sample. Limited DNA yield along with assay requirements often result in insufficient sample to replicate results. This can be especially problematic with archival of samples with unique characteristics. Whole Genome Amplification as a means of robust, highly accurate amplification of small concentrations of DNA is a very attractive tool for increasing the success rate of diagnostic testing.

Alterations in mtDNA have been shown to be potential biomarkers for cancer detection [2,4,24,30]. In this study, we evaluated the utility of Multiple Strand Displacement Whole Genome Amplification as a means of generating large quantities of mtDNA from total DNA without changing the mtDNA sequence due to amplification error or biasing amplification towards a subpopulation. Overall, we found that WGA does amplify nuclear and mtDNA 100 to greater than 10,000 fold and that WGA-amplified mtDNA was of equivalent performance as native mtDNA, with all the disease associated mutations successfully identified in cancer samples with respect to normal controls. Additional low level base transitions were amplified along with the native sequence, appearing as mixtures (heteroplasmic calls). Thus, WGA allows high fidelity, high yield whole genome amplification supporting its use in mitochondrial DNA testing for clinical and research applications when tissue/DNA samples are limiting.

MtDNA sequences obtained from the different versions of the array were compared using the same input DNA. This showed there is no significant difference between sequences obtained from either the version 1 or 2 of the array. The analysis confirmed 6 of 8 sequences to be 100% identical and two samples to have one heteroplasmic difference each resulting in 99.999% sequence identity.

Sequence comparisons between native mtDNA sample and WGA-amplified mtDNA were conducted on eleven samples from non-cancer individuals using the version 1 array. Nine comparisons resulted in no differences between the native and WGA sequences. Confidence in the WGA sequence data was strengthened by the WGA sequence correctly identifying 8 heteroplasmies present in the native sample, demonstrating that bias towards one of the two populations did not occur.

The same results were obtained for blood and tumor DNA from cancer patients as were seen in the non-cancer samples. The sequence comparisons between the eight native mtDNA samples (5 bloods and 3 tumors) and their respective WGA counterparts resulted in a 99.995% correlation between corresponding sequences, similar to the 99.9994% concordance seen in the comparison of native and WGA non-cancer samples. In addition, the number of bases recovered after mtDNA sequencing of WGA DNA was very high: 99.42% for non-cancer samples and 99.23% for cancer samples. In addition the discordance between the callable bases of native and WGA samples ranged from 0.00054% to 0.00495%, which is very comparable to the rate of 0.00328% observed previously for tumor samples sequenced in triplicate on the version 2 microarray [30]. These results demonstrate that the microarray technology used in this study, in conjunction with WGA amplification, should be considered as having the same high performance capabilities as traditional fluorescent based capillary electrophoresis.

## Conclusion

Of the 19 samples examined in this study whole genome amplification by MDA was used to amplify nuclear and mtDNA from clinical samples at a rate of 120 to greater than 10,000 fold. Fold amplification is inversely proportional to the input DNA added to the WGA reaction. The mtDNA sequences of the 19 samples showed 15 instances where the base call of the native sample disagreed with the base call of the matched WGA sample, an event occurring at less than 0.005%. Fourteen of the discrepant calls were heteroplasmic calls, with 13 of 14 being present in the WGA sample sequence and the native sample a homoplasmic base identical to the reference sequence. In all instances, the heteroplasmy in the WGA sample was a transition; containing the same base identified in the native sample as well as the other purine or pyrimidine. There was a single instance of a heteroplasmy being present in the native sample, but absent from the WGA material (Table 2). It is unclear whether or not the heteroplasmies detected after WGA were present in the native sample in very low abundance and below the detection limit of the sequencing technology, or if these populations were introduced erroneously by the WGA technology. Heteroplasmies detected in samples that have been pre-amplified by WGA should therefore be carefully examined to confirm their authenticity.

## Competing interests

The author(s) declare that they have no competing interests.

## Authors' contributions

SM preformed the technical lab work. SM, CDO, and JPJ performed data analysis and drafted the manuscript. WNR and DS provided the clinical samples and clinical expertise. PDW and SS provided funding and project leadership. All authors have read and approved this manuscript.

## Pre-publication history

The pre-publication history for this paper can be accessed here:


